# Engineering Isoprenoid Biosynthesis in *Artemisia annua* L. for the Production of Taxadiene: A Key Intermediate of Taxol

**DOI:** 10.1155/2015/504932

**Published:** 2015-02-01

**Authors:** Meiya Li, Fusheng Jiang, Xiangli Yu, Zhiqi Miao

**Affiliations:** ^1^Fudan-SJTU-Nottingham Plant Biotechnology R&D Center, School of Agriculture and Biology, Shanghai Jiao Tong University, Shanghai 200240, China; ^2^Institute of Biotechnology, College of Life Science, Zhejiang Chinese Medical University, Hangzhou 310053, China

## Abstract

Taxadiene is the first committed precursor to paclitaxel, marketed as Taxol, arguably the most important anticancer agent against ovarian and breast cancer. In *Taxus*, taxadiene is directly synthesized from geranylgeranyl diphosphate (GGPP) that is the common precursor for diterpenoids and is found in most plants and microbes. In this study, *Artemisia annua* L., a Chinese medicinal herb that grows fast and is rich in terpenoids, was used as a genetic engineering host to produce taxadiene. The *TXS* (taxadiene synthase) gene, cloned from *Taxus* and inserted into pCAMBIA1304, was transformed into *Artemisia annua* L. using the *Agrobacterium tumefaciens*-mediated method. Thirty independent transgenic plants were obtained, and GC-MS analysis was used to confirm that taxadiene was produced and accumulated up to 129.7 *μ*g/g dry mass. However, the high expression of *TXS* did not affect plant growth or photosynthesis in transgenic *Artemisia annua* L. It is notable that artemisinin is produced and stored in leaves and most taxadiene accumulated in the stem of transgenic *Artemisia annua* L., suggesting a new way to produce two important compounds in one transgenic plant: leaves for artemisinin and stem for taxadiene. Overall, this study demonstrates that genetic engineering of the taxane biosynthetic pathway in *Artemisia annua* L. for the production of taxadiene is feasible.

## 1. Introduction

Taxanes are a class of polycyclic diterpenes produced by many species of yew. Among these taxanes, paclitaxel, also known as Taxol, is the most important chemotherapy drug in the treatment of ovarian and breast cancers and diseased vasculature [1, 2]. The annual sale of Taxol and its analogue Taxotere reached up to 3.7 billion in 2006. However, only limited amounts can be obtained from the currently available sources. Total synthesis of Taxol was successfully finished in 1994; however, the complex structure of Taxol results in too low yield to be cost effective [3]. Semisynthesis methods start from more abundant and readily available precursors, such as 10-deacetylbaccatin III and baccatin III from* Taxus,* but the limited availability of natural yew trees, slow growth rate of cultivated ones, and the low yield of taxanes result in a high price for Taxol [4]. Meanwhile, excessive exploitation of wild trees creates environmental damage and has been prohibited in many countries. It is considered that cell cultures and entophytic fungi fermentation are good sources of Taxol or its intermediates [5–7]; however, the processes of cell culture are not easy and yield is low.

Metabolic engineering is a useful strategy for production of natural product in plant, such as producing tanshinone in* Salvia miltiorrhiza* hairy root cultures [8] and enhancing the production of tropane alkaloids in transgenic* Anisodus acutangulus *hairy root cultures [9]. Taxadiene is the first taxane in the pathway of Taxol biosynthesis, which is synthesized from geranylgeranyl diphosphate (GGPP) directly. Taxadiene synthase, located in plastids, catalyzes the cyclization of GGPP to taxadiene [10, 11]. Because no routine transformation systems are currently available for* Taxus* species, researchers have explored the possibility of transferring the pathway to microbial or fast-growing plant species that are easier to genetically manipulate to produce taxoids by metabolic engineering. By expressing GGPP synthase and a truncated taxadiene synthase gene, taxadiene can be synthesized in* Escherichia coli* [12]. Further, eight Taxol biosynthetic genes were transformed and expressed in* Saccharomyces cerevisiae* to obtain advanced taxanes, such as taxadiene-5*α*-acetoxy-10*β*-ol [13]. Because the universal diterpenoid progenitor, GGPP, is rich in most plants, syntheses of taxanes from transgenic plants, such as* Arabidopsis *[14] and tomato [15], have been investigated. However, constitutive expression of taxadiene synthase caused concomitant growth retardation and reduced levels of photosynthetic pigments in both plant systems. But in ginseng roots [16], the expression of taxadiene synthase gene did not affect the phenotype or growth.

**Figure 1:**
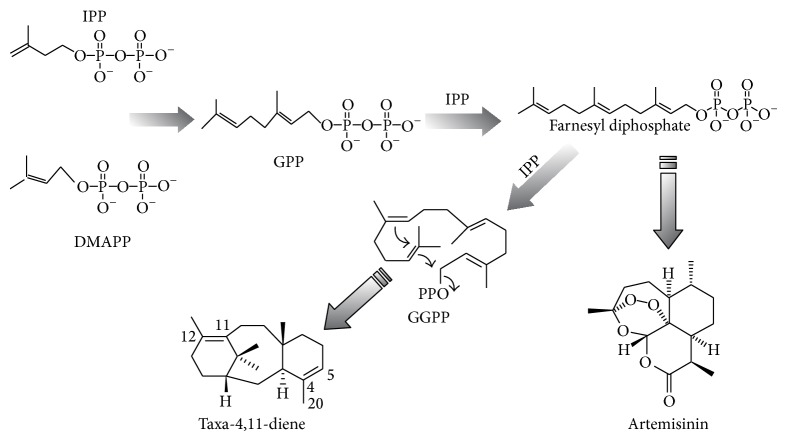
The biosynthetic pathway of secondary metabolites, including taxadiene and paclitaxel in transgenic* A*.* annua*.

In this study,* Artemisia annua *L., a Chinese annual herb, was engineered to produce taxadiene for several reasons.* A. annua *L. has an abundant amount of terpene precursors, such as IPP and FPP, with total terpenoids in leaves recorded up to 3% of the dry mass [17]. As a traditional medical plant [18] which has been cultured in China for many years,* A. annua* L. grows fast, can reach more than 1.8 m in height, and has a high yield of biomass. Moreover, an efficient genetic transformation system has been established for* A. annua* L. To regulate the metabolism of terpenoid, divert some GGPP to taxadiene biosynthesis and construct a new system to produce taxadiene and even some more advanced taxanes (Figure 1); the taxadiene synthase gene was transformed into* A. annua.*

## 2. Materials and Methods 

### 2.1. Transformation of* Artemisia annua* L

Seeds of* A. annua.* were collected from Youyang, Chongqing, China. The seeds were surface-sterilized in 75% ethanol for 1 min followed by treatment with 20% (v/v) sodium hypochlorite (NaOCl) for 20 min, washed 3 to 4 times with sterile distilled water, and sown onto MS_0_ medium [Murashige and Skoog (MS) basal medium supplemented with sucrose (30 g/L) and phytoagar (Sigma) (2.6 g/L)] [19] in Petri dishes (9 cm diameter). Plants were grown with a 16 h light/8 h dark photoperiod and 8,000 Lux (metal halide source) of light at 25°C. When reaching 5 cm in height, germinated seedlings were collected and the leaves were cut into 0.5 cm diameter pieces and used as the explants in* Agrobacterium tumefaciens*-mediated leaf disc transformation [20]. The plant binary expression vector pCAMBIA1304::p35s-*TXS*-nos, derived from pCAMBIA1304 in which the GUS reporter gene was replaced with the* TXS* gene, was used for transformation into* Agrobacterium tumefaciens *strain EHA105.

After coculture in MS_0_ and hygromycin selection in MS_1_ (MS_0_ + 0.5 mg/L 6-BA + 0.05 mg/L NAA + 10 mg/L hygromycin + 500 mg/L carbenicillin), the hygromycin-resistant shoots were regenerated and transferred to rooting medium MS_2_ (1/2 MS_0_ + 5 mg/L hygromycin + 250 mg/L carbenicillin) [20]. After roots were formed (about 14–21 d), the rooted plantlets were transferred to soil and grown under controlled conditions in the growth chamber. After 2-3 months, transformed plants were transplanted to the field and covered by plastic film for further growth.

### 2.2. PCR Analysis of* TXS* and* hpt*II Genes in Transgenic Plants

The taxadiene synthase gene (*TXS*) from transgenic* A. annua* plants, which were about 10 cm in height, was identified by PCR analysis using genomic DNA isolated by the CTAB method [21]. Primers p35S (5′-GAT GAC GCA CAA TCC CAC T-3′) and TXSR (5′-CGT TTC GTG AGA GTT CTA CTT ACC-3′) were used to identify the introduced* TXS* gene, and primers* hpt*IIF (5′-GAC ATT GTT GGA GCC GAA A-3′) and* hpt*IIR (5′-TGC TTG ACA TTG GGG AGT T-3′) amplified the* hpt*II gene. The PCR reaction was carried out using the following mixture [forward primer (10 *μ*M) 0.5 *μ*L, reverse primer (10 *μ*M) 0.5 *μ*L, 10 × PCR buffer 2.5 *μ*L, dNTPs (2.5 mM) 2.0 *μ*L, MgCl_2_ (25 mM) 1.5 *μ*L, DNA Template (100 ng/*μ*L) 2.0 *μ*L,* Taq* enzyme (5 U/*μ*L) 0.2 *μ*L, add ddH_2_O up to 25 *μ*L] under the following conditions: 94°C for 5 min followed by 32 cycles of amplification (94°C for 45 s, 58°C for 45 s, and 72°C for 45 s) and 72°C for 10 min, resulting in 567 bp and 383 bp DNA fragments for the* TXS *and* hpt*II genes, respectively.

### 2.3. DNA Isolation and Southern Blot Analysis

Genomic DNA from transgenic and control plants were isolated and purified according to the CTAB method. Approximately 40 *μ*g of DNA per sample was digested with* Hin*dIII, fractionated by 1.0% agarose gel electrophoresis, transferred onto a positively charged Hybond-N^+^ nylon membrane (Amersham Biosciences, UK), and hybridized with an alkaline phosphatase-labeled partial* hpt*II sequence as the probe. The probe (383 bp) was generated by PCR using* hpt*II as the template, with primers* hpt*IIF1 and* hpt*IIR1. Probe labeling (alkaline phosphatase), hybridization, and signal detection were performed using Amersham AlkPhos Direct Labeling Reagents (GE Healthcare, UK) and the CDP-Star Detection Module following the manufacturer's instructions (Amersham Biosciences, UK). The hybridized signals were visualized by exposure to Fuji X-ray film at room temperature for 2-3 h.

### 2.4. RNA Isolation and Semiquantitative RT-PCR Analysis

RNA was extracted from upper leaves of control and transgenic* A. annua* plants using the RNAprep Plant Kit [TIANGEN Biotech (Beijing) Co., Ltd.]. DNA contamination was removed with DNaseI (TaKaRa) following the protocol provided by the manufacturer. The cDNA synthesis was completed from the RNA samples using reverse transcriptase reagent (TaKaRa) according to the manufacturer's instructions.

A 291 bp fragment of the* TXS *gene (corresponding to* TXS* cDNA from nucleotides 335 to 626) was prepared by PCR amplification from cDNA with the following primers: TSF1 (5′-CGT TTC GTG AGA GTT CTA CTT ACC-3′) and TSR1 (5′-CCG AGA GGG CGA TAA CAG A-3′). The gene-specific primers for ubiquitin conjugation (UBC) gene are UBC-F (5′-CAC ACT TGA GGT TGA GTC CAG-3′) and UBC-R (5′-CAT AAC ATT TGC GGC AGA TAG-3′). The PCR reaction was carried out using the following mixture [forward primer (10 *μ*M) 0.5 *μ*L, reverse primer (10 *μ*M) 0.5 *μ*L, 10 × PCR buffer 2.5 *μ*L, dNTPs (2.5 mM) 2.0 *μ*L, MgCl_2_ (25 mM) 1.5 *μ*L, DNA Template (100 ng/*μ*L) 2.0 *μ*L,* Taq* enzyme (5 U/*μ*L) 0.2 *μ*L, add ddH_2_O up to 25 *μ*L] under the following conditions: 94°C for 5 min followed by 35 cycles of amplification (94°C for 30 s, 58°C for 30 s, and 72°C for 30 s) and 72°C for 10 min.

Following the separation of the PCR products on ethidium bromide stained 1% agarose gels, the bands at 291 bp were quantified. Each band was normalized against the intensity of the UBC control band obtained with the same cDNA sample.

### 2.5. Quantification of Taxadiene by Gas Chromatography-Mass Spectrometry (GC-MS)

To detect the new taxanes arising after the transformation of* TXS* gene-taxadiene, leaves of 3 independent transgenic* A. annua* plants and a wild control plant were collected after growing in the field for about 2.5 months and 6.5 months. Leaves were weighed and milled to a fine power in liquid N_2_, followed by addition of 15 mL of hexane and treatment with ultrasonication for 30 min at 75 Hz. The extract was filtered, and the plant leaves were reextracted twice more according to the same method. These extracts were pooled and concentrated to 5 mL and then washed through a small (1 × 5 cm) silica gel column. The hexane elute was collected and concentrated to 1 mL.

1 *μ*L of the hexane solution was analyzed by GC-MS (Perkin Elmer, Auto System XL GC/Turbo Mass MS) equipped with the capillary column DB-5MS (30 m × 0.25 mm × 0.25 *μ*m) under the following condition: injection port temperature, 285°C; carrier gas, He (99.999%) 1.0 mL min^−1^; no split ratio; the column temperature increase at a speed of 10°C/min from 80°C to 300°C after a 2 min delay and then maintenance for 16 min at 300°C; electron ionization voltage, 70 eV. The taxadiene was analyzed according to NIST98 and WILEY7.0 mass spectrum database, by comparison to a nonadecane (Sigma) internal standard (1 *μ*g/mL), to estimate total taxadiene content.

### 2.6. Quantification of Artemisinin Using High Performance Liquid Chromatography-Evaporative Light Scattering Detection (HPLC-ELSD)

Leaves of* A. annua* collected as described above were dried at 45°C and ground. The leaf powder (0.1 g/sample) was extracted with ethanol (1 mL) by ultrasonication (twice, 15 min each, 75 Hz), and then centrifuged for 10 min at 12,000 rpm to remove the suspended particles. The final supernatant was filtered through a 0.22 *μ*m filter.

The prepared samples were analyzed by Waters Alliance 2695 HPLC system coupled with Waters 2420 ELSD detector. The HPLC condition was Waters C_18_ column using water : methanol (40 : 60, v/v) mixture as a mobile phase at a flow rate of 1 mL/min. The ELSD condition was optimized at a nebulizer-gas pressure of 50 psi and drift tube temperature of 45°C, and the gain was set at 7. The authentic artemisinin purchased from Sigma (St. Louis, USA) was used as the standard control. For each sample, the injection volume was 20 *μ*L, and the results were analyzed with the Empower data system.

### 2.7. Observation of the Growth Influence of* TXS* on Transgenic and Wild Plants

To investigate the influence of transformed* TXS* gene on the growth of* A. annua*, transgenic and wild plants were grown in the greenhouse for two months and then transplanted to the field for another eight months. After about 2.5 months, the height, number of branch stems, and diameter of the stalk were measured. After collecting the seeds, whole plants were weighed and a thousand seeds from different plants were germinated to compare the germination capacity.

## 3. Results and Discussion

**Figure 2:**
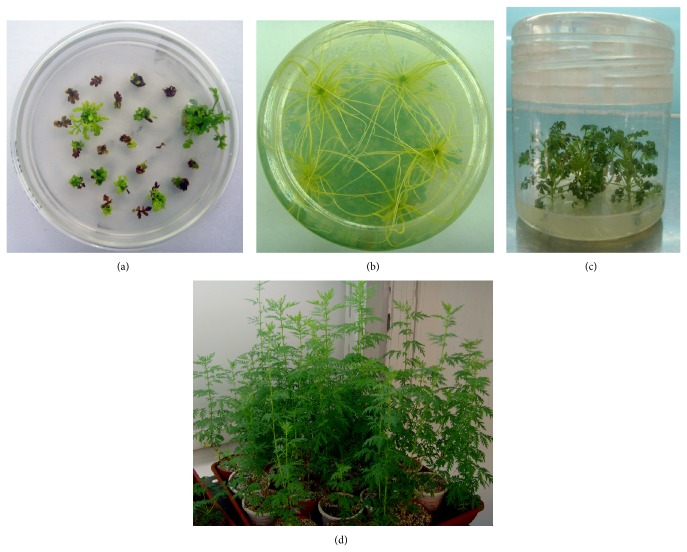
Pictures of* A. annua* at different stages of transformation and regeneration. (a) Screening; (b) growing in the bottle; (c) shoot regeneration; (d) growing in the greenhouse.

**Figure 3:**
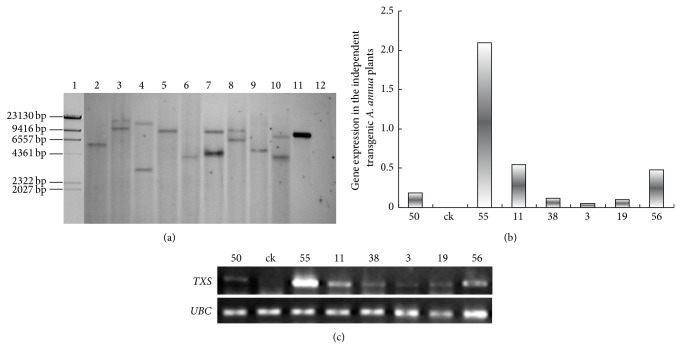
Southern blot and semiquantitative RT-PCR analysis of transgenic* A*.* annua. *(a) Southern blot analysis of* A*.* annua* transformed with pCAMBIA1304::p35s-*TXS*-nos. Lane assignment: 1 *λ*/*Hind*III DNA molecular marker; 2–10 genomic DNA isolated from independent transgenic* A*.* annua* lines 3, 9, 11, 55, 56, 19, 21, 38, and 50; 11 pCAMBIA1304-*TXS*; 12 genomic DNA of untransformed wildtype* A*.* annua* (b) and (c) semiquantitative RT-PCR analysis for the expression of* TXS* in independent transgenic* A*.* annua* lines 50, 55, 11, 38, 3, 19, and 56. ck indicates the untransformed wildtype control* A*.* annua*; UBC: ubiquitin conjugation gene;* TXS*: taxadiene synthase gene.

EHA105 harboring the plasmid pCAMBIA1304::p35s-*TXS*-nos was used to introduce the* TXS* gene into the young leaves of* A*.* annua *via* A. tumefaciens*-mediated transformation. After hygromycin selection and regeneration, 67 independent hygromycin-resistant plantlets were obtained (Figure 2), and among them 40 independent transgenic plants were confirmed to have the* TXS *and* hpt*II genes by PCR. The consistent presence of the* TXS *and* hpt*II genes indicated that both were transformed into plant cells as an intact T-DNA sequence. Therefore, the* TXS* copy number can be determined by an* hpt*II gene probe in Southern hybridization. The probe hybridized to the supercoiled pCAMBIA1304::p35s-*TXS*-nos but did not hybridize to wild* A*.* annua*. Young plant leaves from the transgenic T1 generation were harvested and checked for* TXS* gene insertion by Southern blot analysis. The results confirmed the T-DNA integration in all independent lines of transgenic* A*.* annua*. Single bands were detected in four lines (3, 50, 55, and 56) and double bands in five lines (9, 11, 19, 21, and 38) (Figure 3(a)). The transgenic lines with less than two* TXS* gene copies were expected to express the transgene at a higher level, and the following experiments were focused on these lines.

The* TXS* gene expression in young leaves of transgenic lines was further analyzed by semiquantitative RT-PCR. A housekeeping gene,* UBC*, was used as a reference to normalize the transgene expression level.* TXS* expression was detected in approximately 30 transgenic lines; however, there were significant differences in* TXS* expression between lines (Figures 3(b)/3(c)). The highest* TXS* expression was detected in transgenic line 55, which was approximately 3 times higher than that in lines 11 and 56 and contained only one transgene copy.

**Figure 4:**
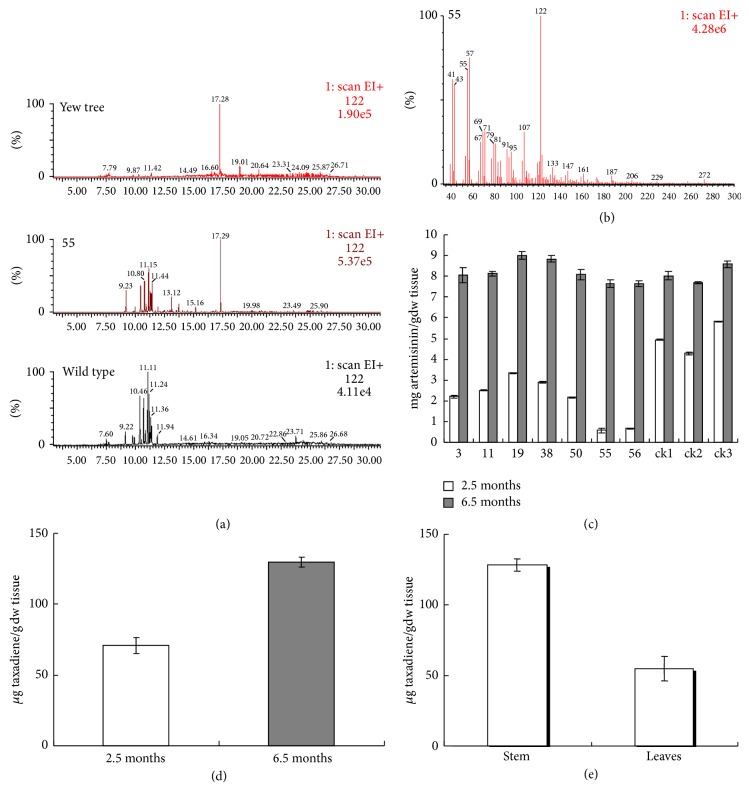
(a) GC-MS analysis for taxadiene in the crude hexane extracts from yew and transgenic and wildtype* A*.* annua*. The GC profiles of the ion current at* m/z* 122 from hexane extracts of a yew tree, the* A*.* annua* transgenic line 55, and the wildtype control of* A*.* annua*. (b) The mass spectrum of the peak with retention time of 17.29 min, matching the published MS data of taxadiene. (c) Artemisinin analysis in transgenic and untransformed* A*.* annua* plants by HPLC-ELSD. Plants that were grown in the field for 2.5 and 6.5 months, respectively, were harvested to determine the content of artemisinin in wild control and transgenic* A*.* annua*. ck: the wildtype; 3, 5, 11, 19, 30, 55, and 56: independent transgenic lines of* A*.* annua*. (mean ± SD, *n* = 3). (d) The amount of taxadiene in 2.5- and 6.5-month-old* A*.* annua* transgenic line 55 (mean ± SD, *n* = 3). (e) Taxadiene content in the stem and the leaves of 6.5-month-old* A*.* annua* transgenic line 55 (mean ± SD, *n* = 3).

Taxadiene was purified from hexane extracts of lines 55, 56, and 11 using a silica gel column and then analyzed by GC-MS. The ion flow data at* m/z* 122, reported as the most abundant broken ion of taxadiene [10], was used for identifying and quantifying taxadiene in hexane extracts. The data showed that the hexane extracts from all three transgenic lines gave a GC-MS peak at 17.29 min that was absent from the wild control (Figure 4(a)). The major mass spectrum ions were at* m/z* 122, 121, 123, 107, and 272, which is consistent with the published data on taxadiene [22] (Figure 4(b)). A similar peak was also detected in the yew hexane extracts following the same preparation. Together these results indicated that the transgenic* A*.* annua *is synthesizing taxadiene. Using nonadecane (Sigma, Cat. 39756-36-0) as an internal standard, the contents of taxadiene were estimated from 4.4 to 129.7 *μ*g/g dry weight in the transgenic* A*.* annua* lines. In transformed tomato with* TXS* gene, the contents of taxadiene was 20 *μ*g/g dry weight [15] and in transgenic ginseng roots was 9.1 *μ*g/g dry weight [16]; the highest taxadiene yield in transgenic* A*.* annua* was gotten from line 55, since the expression of the* TXS* gene in line 55 is much higher than others, which may indicate that the higher the gene expressed, the more the taxadiene produced.

Artemisinin is the main terpenoid in wild* A*.* annua*. However, most IPP, the universal terpenoid building block, in transgenic* A*.* annua* is diverted into the taxadiene pathway as the result of the introduction of taxadiene synthase. Therefore the biosynthesis of artemisinin may be competitively inhibited in transgenic* A*.* annua.* To investigate the interaction between the biosynthesis of taxadiene and artemisinin in transgenic* A*.* annua*, the contents of artemisinin in transgenic and wild lines of* A*.* annua* were measured by HPLC-ELSD. The results revealed that transgenic lines 55 and 56 produced more taxadiene and less artemisinin after growing in the field for 2.5 months: the artemisinin content was 0.057% in line 55 and 0.066% in line 56, while the content of artemisinin reaches up to 0.58% in the wild control (Figure 4(c)). Since FPP is the common precursor to taxadiene and artemisinin and its content is limited in the 2.5-month-old transgenic* A*.* annua*, the correlation between the decrease of artemisinin and the increase of taxadiene suggests that the introduction of taxadiene synthase changed the FPP metabolic flux. It is likely that more FPP is conscribed to produce GGPP, some of which was further bound to taxadiene synthase and converted into taxadiene, which decreased the biosynthesis of artemisinin. However, more FPP is produced in* A*.* annua* after growing in the field for 6.5 months andwhen it is blooming. At this stage, the transgenic* A*.* annua* produced more taxadiene than, but a similar amount of artemisinin to, the wildtype when it is blooming (Figures 4(c)/4(d)).

It is reported that artemisinin is produced by the trichome cell in the leaves, floral buds, and flowers of* A*.* annua* but not in the stem [23, 24]. Usually the leaves of* A*.* annua* are harvested for extracting artemisinin and the stems are discarded as waste. Our research shows that the content of taxadiene in the stems is much higher than that in the leaves of transgenic* A*.* annua*, indicating that transgenic* A*.* annua* can be used for producing taxadiene in the stems and artemisinin in the leaves. It was also found that, in transgenic* A*.* annua* plants, taxadiene accumulated during growth and reached the highest level in the late growth stage, especially when the plant is blooming (Figure 4(e)). Therefore, harvesting transgenic* A*.* annua* in bloom is an efficient way to obtain the highest content of artemisinin and taxadiene at the same time.

**Figure 5:**
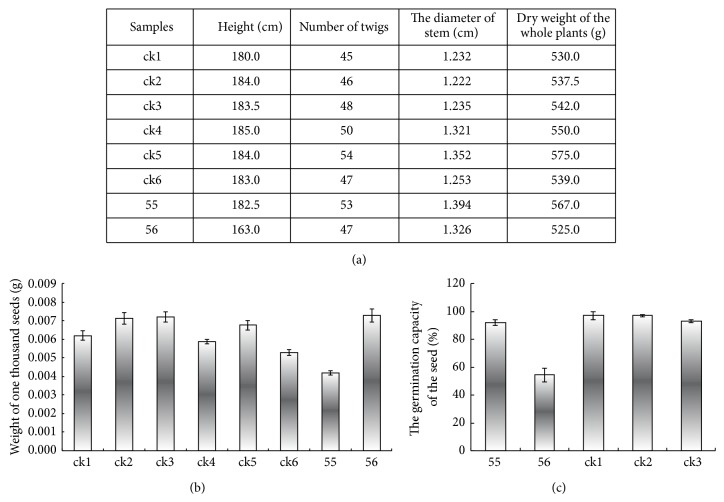
(a) Comparison of the height, number of twigs, stem diameter, and whole plant dry weight between transgenic lines 55 and 56, expressing high levels of* TXS*, and the wildtype control lines of* A*.* annua *(ck1–6). (b) The weight of a thousand seeds of transgenic and wild* A*.* annua* plants (mean ± SD, *n* = 3). ck1–6: different wildtype plants; 55 and 56: independent transgenic* A*.* annua*. (c) The germination capacity (%) of different seeds (mean ± SD, *n* = 3). ck1–3: three different wildtype lines of* A*.* annua*; 55 and 56: lines of independent transgenic* A*.* annua*.

A strong growth retardation and photosynthesis inhibition resulted from high expression of* TXS* in transgenic* Arabidopsis* [14] and tomato [15], but this was not observed in transgenic ginseng roots [16]; it says that, in leaves and stems, most GGPPs could convert to taxadiene, so they will produce less gibberellin, which made the transgenic plants growth retardate, but for roots, they do not need gibberellins for grow. In our research, no phenotypic or growth differences were observed in transgenic* A*.* annua*. In the growth chamber and greenhouse, all the plants in pots grew with similar height and the leaves were green and healthy. After growing in the field for 2.5 months, the height, branching, and stem diameters of both wild and transgenic* A. annua* were measured (Figure 5(a)). All plants grew well, healthy, and strong, suggesting that expression of the introduced* TXS *did not affect* A*.* annua* growth. Additionally, whole plants were dried and weighed after collecting seeds, but no significant difference was found between the masses in different plant lines (Figure 5(a)). One possibility is that there is a more effective biosynthesis system for terpenoid in* A. annua*. In this case, the terpenoid building blocks, such as FPP and GGPP, are abundant enough to produce important primary substances, such as chlorophylls, carotenoids, gibberellins, and tocopherols, in transgenic* A*.* annua*, even when some GGPP is consumed by the taxadiene synthase, so the biosynthesis of taxadiene does not strongly influence the content of terpenoid precursor in* A*.* annua* leaves. This is supported by the results that the taxadiene content is much higher in the stem than in the leaves in transgenic line 55 (Figure 4(e)).

The only disadvantage of high* TXS* expression in* A*.* annua* is that the seeds became smaller and lighter (Figure 5(b)). However, the reduction in seed size and weight did not influence germination (Figure 5(c)). It is unclear why the seed size of the transgenic plant is smaller.

## 4. Conclusion

Our study demonstrates that genetic engineering of isoprenoid biosynthesis to produce taxadiene in* A*.* annua* is feasible. The transformation of* A*.* annua* with the* TXS* gene did not result in growth retardation or photosynthesis inhibition. It is notable that the content of taxadiene reached up to 129.7 *μ*g/g dry weight in transgenic* A*.* annua* line 55 and mostly concentrates in stems, while artemisinin accumulates in leaves. Therefore, transgenic* A*.* annua* harboring the* TXS* gene can be used to produce both artemisinin and taxadiene: leaves for artemisinin and stems for taxadiene.

In future studies, more Taxol biosynthesis genes, including taxane 2*α*-hydroxylase, 5*α*-hydroxylase, 7*β*-hydroxylase, and 10*β*-hydroxylase, will be transformed into* A*.* annua* to produce more advanced taxoids that could be purified and used for semisynthesis of Taxol and Taxotere. Furthermore, novel taxanes arising as the result of transgenic genes in* Taxus* and those encoded by* A*.* annua* itself will be monitored.
